# How I manage TFCC injuries

**DOI:** 10.1186/1753-6561-9-S3-A44

**Published:** 2015-05-19

**Authors:** Toshiyasu Nakamura

**Affiliations:** 1 Department of Orthopaedic Surgery, Sanno Hospital, 107-0052, Tokyo, Japan

## 

The TFCC consists of triangular fibrocartilage (TFC), meniscus homologue, ulnolunate ligament, ulnotriquetral ligament, radioulnar ligament, and sheath floor of the extensor carpi ulnaris (ECU). The TFCC functions as a stabilizer of the ulnocarpal and DRU joints, load distributor between ulna and ulnar carpus and introduces smooth forearm rotation. The TFCC is a hammock-like shape supporting and surrounding the ulnar carpus, with the strong and nearly vertical anchor to the ulnar fovea, i.e. the RUL. During forearm rotation, the radius, the carpus and distal surface of the TFCC rotate over the ulnar head, thus simple twisting of the RUL occurs at its foveal attachment. The main stabilizer of this tiny joint is the proximal component of the TFCC, the radioulnar ligament (RUL). Central attaching fibers of the RUL at the fovea indicate isometric pattern during forearm rotation, while eccentric fibers at the fovea and base of the styloid indicate changes in length, dorsal indicates longer length in pronation, while palmar indicates longer in supination. When the RUL is torn, instability of the DRUJ occurs. The greater the tear of the RUL be, the greater instability of the DRUJ indicates. When the RUL is ruptured at the fovea, the RUL must be introduced into the central lesion of the fovea of the ulna. Classical Palmer 1B peripheral lesion also demonstrates moderate DRUJ instability, because this lesion is close to the RUL. Other traumatic lesions on the TFCC, such as Palmer 1A and 1D tear, did not indicate DRUJ instability.

Diagnosis of DRUJ instability due to TFCC tear is achieved by physical examination, such as DRUJ ballottement test, arthrogram, MRI and arthroscopy. Image diagnosis is useful, however arthroscopy is a gold standard for diagnosis as well as treatment of the TFCC lesions. DRUJ arthroscopy, recently introduced arthroscopic technique, provides direct visualization of fovea lesion, where the RUL attaches.

Treatments of TFCC lesions are depending on the tear site. Palmer 1B lesion is easily treated by arthroscopic capsular repair. Debridement is useful for palmer 1A or 1D tear. We also prefer to shorten the ulna, when there were degenerative changes on the TFC (Palmer Class 2) or with ulnar positive variance. Treatment options for DRUJ instability due to TFCC foveal avulsion are arthroscopic and open repair of the TFCC to the fovea for acute or sub-acute cases and reconstruction of the TFCC using ECU half-slip tendon or PL tendon for chronic TFCC injuries. Ulnar shortening procedure can be useful for partial avulsion of the RUL either dorsal or palmar, but not for complete avulsion of the TFCC at the fovea. Arthroscopic partial resection of the TFCC can no longer indicated for unstable DRUJ, because it does not introduce stabilization of the DRUJ.

